# Characteristics of Sports-Related Emergency Transport: A Population-Based Descriptive Study in Osaka City

**DOI:** 10.2188/jea.JE20190019

**Published:** 2020-06-05

**Authors:** Kosuke Kiyohara, Junya Sado, Tasuku Matsuyama, Yusuke Katayama, Sumito Hayashida, Ken Nakata, Tetsuhisa Kitamura

**Affiliations:** 1 Department of Food Science, Faculty of Home Economics, Otsuma Women’s University, Tokyo, Japan; 2 Medicine for Sports and Performing Arts, Department of Health and Sport Sciences, Graduate School of Medicine, Osaka University, Osaka, Japan; 3 Department of Emergency Medicine, Kyoto Prefectural University of Medicine, Kyoto, Japan; 4 Department of Traumatology and Acute Critical Medicine, Osaka University Graduate School of Medicine, Osaka, Japan; 5 Osaka Municipal Fire Department, Osaka, Japan; 6 Division of Environmental Medicine and Population Sciences, Department of Social and Environmental Medicine, Graduate School of Medicine, Osaka University, Osaka, Japan

**Keywords:** emergency transport, sports, ambulance records

## Abstract

**Background:**

Little is known about the characteristics of emergency patients transported to hospital while participating in sports activity. Hence, we identified characteristics of emergency patients transported to hospital by emergency medical service (EMS) while participating in sports activity in Osaka City.

**Methods:**

Population-based ambulance records of Osaka Municipal Fire Department were reviewed. All sports-related emergency transport cases (ie, patients experiencing external injury or illness during/immediately after participation in sports activity and then transported to hospital by the EMS) were enrolled, including both athletes and recreational sports participants. The study was performed from January 1, 2013 to December 31, 2015. Data of patient characteristics were described according to the type of sports.

**Results:**

During the study, 661,190 patients required emergency transport in Osaka city; 2,642 (0.4%) were sports-related emergency transport, including 2,453 external injuries and 298 illnesses. Overall, 79.0% of patients were men and 44.4% were less than 18 years. Emergency transport during ball games accounts for the majority of cases (71.5%, 1,888/2,642), including baseball (*n* = 380), soccer (*n* = 368), and futsal (*n* = 209). The leading diagnosis/symptom of external injury was fracture/bone contusion (*n* = 701) and that of illness was heatstroke/dehydration (*n* = 184). Serious acute illness, such as sudden cardiac arrest, accounted for 0.6% (16/2,751) of all accidents, with half of them (*n* = 8) related to long-distance running.

**Conclusion:**

Characteristics of sports-related accidents widely varied by type of sports. Measures to prevent serious accidents during sports activities should be established based on the information on patient characteristics of each type of sports.

## INTRODUCTION

Although the proportion of the general adult population engaging in sports activities has been increasing in the last 30 years in Japan,^1^ participation in sports sometimes leads to injuries and acute illnesses. In particular, serious injuries and illnesses during sports activities that result in emergency transportation to a hospital are important problems for both competitive athletes and recreational sports participants, affecting daily life and the player’s career. Therefore, an evidence-based strategy should be implemented to prevent serious accidents during sports activities. Improved understanding of the epidemiological features of cases requiring emergency transport could be instrumental in planning appropriate preventative strategies in the community setting. However, currently, the epidemiology of sports-related emergency transport has not been sufficiently investigated at the population level. Hence, in the present study, using the population-based ambulance records in Osaka City in Japan, we aimed to reveal the detailed characteristics of emergency patients transported to hospital via the emergency medical service (EMS) when they were participating in sports activities.

## METHODS

### Study design and participants

This was a retrospective observational study performed using exhaustive ambulance records in Osaka City, the largest metropolitan city in western Japan, with a population of approximately 2.7 million in 2015, covering an area of 222 km^2^. The study period was 3 years, from January 1, 2013 to December 31, 2015. All sports-related emergency transport cases registered in ambulance records during this period were enrolled (ie, both competitive athletes and recreational sports participants were included). Herein, “sports-related emergency transport” indicates patients with external injury or illness during/immediately after participation in sports activity and then transported to hospital via the EMS. Patients who had missing information regarding the type of sports in which they participated were excluded from the analysis.

### Emergency medical service system in Osaka City

The EMS is a public service, and patients are transported to hospital via the public EMS system. In 2015, there were 25 fire stations with 60 ambulances and one dispatch center in Osaka City. The EMS system is operated by the Osaka Municipal Fire Department and is activated by phoning 119. EMS life support is provided 24 h a day, 7 days a week. Usually, each ambulance has a crew of three emergency providers, including at least one emergency lifesaving technician who is a highly-trained prehospital emergency care provider authorized to use an automated external defibrillator, to insert an intravenous line and administer adrenaline, and to place advanced airway management. The annual number of patients transported to hospitals by the EMS in this area is approximately 200,000. In Osaka City, emergency dispatchers do not make phone calls to hospitals to determine patient acceptance. Instead, using the protocol established by the Osaka Municipal Fire Department, EMS ambulance crews at the scene select the appropriate medical institutions near the scene that are best able to treat emergency patients according to the urgency or the patient’s symptoms.

### Quality control of data

EMS personnel record individual patient data in cooperation with the physicians caring for the patients and then transfer the data to the information center in the Osaka Municipal Fire Department. If the data sheet was incomplete, information center personnel returned it to the responsible EMS personnel so that they could complete it.

### Data collection

Data of sports-related emergency transport cases were uniformly collected using ambulance records in Osaka City, including the date and time of occurrence, age, sex, and location of emergency call. In addition, the type of sports, mechanism of accident, body part with injury/sickness, and diagnosis/symptoms were determined by four investigators by reviewing the detailed descriptions of each accident, which are filled by EMS personnel based on the situations at the scene and patients’ and/or bystanders’ interviews. As for the mechanism of accident, the event that occurred earliest was chosen as the cause of injury/sickness. The information on diagnosis/symptoms and body parts with injuries and illnesses was classified based on a previous study.^2^

### Statistical analysis

Summary statistics were expressed as the mean and standard deviation for numerical variables and as proportions for categorical variables. The age categories and the diagnosis/symptoms of external injury and illness were counted according to the type of sports. The number of patients considering the age and body parts affected by the type of sports is shown in eTable 1 and eTable 2. Here, when a patient suffered two or more injuries/illnesses in an accident (eg, a person suffered patella fracture and knee ligament rupture at the same time), each injury/illness was separately counted. Statistical analyses were performed using SPSS statistical package V.25.0J (IBM Corp. Armonk, NY, USA).

### Ethics

This study was approved by the Ethics Committees of Osaka University Graduate School of Medicine and Kyoto University Graduate School of Medicine. Personal identifiers were previously removed from the database by EMS personnel; therefore, the requirement for written informed consent from each patient was waived.

## RESULTS

**Figure 1.**
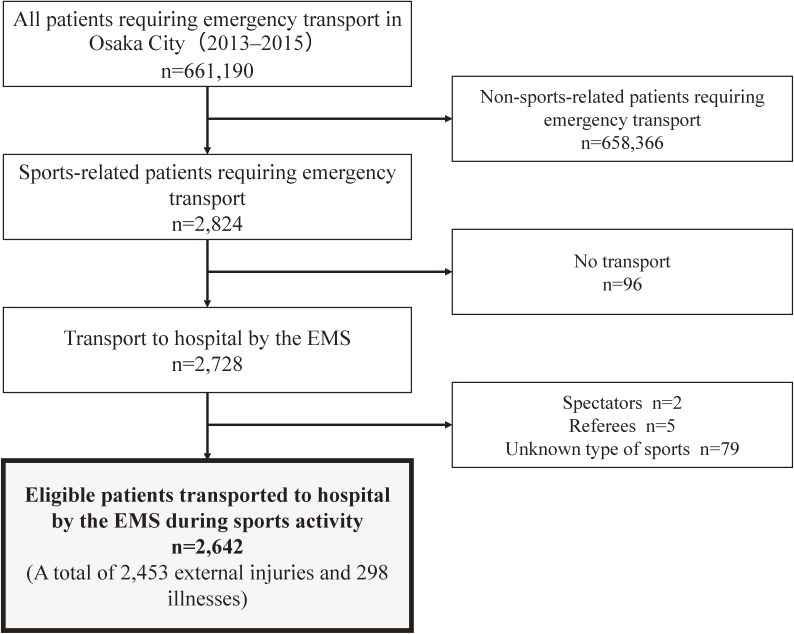
Flowchart showing the selection of patients who required sports-related emergency transport in Osaka City (2013–2015)

Figure 1 shows the flowchart for the selection of eligible patients for analysis. During the 3-year study period, there were 661,190 patients who required emergency transport in Osaka City, and 2,642 of them (0.4%) were transported to hospital owing to sports-related accidents. Table 1 shows the characteristics of patients who required sports-related emergency transport. Overall, 79.0% of patients were male and 44.4% were younger than 18 years. The sports-related accidents most commonly occurred in autumn (30.1%), and slightly increased from 2013 to 2015. Most accidents were due to external injury (89.6%) and the most common cause of injury was falling (33.9%).

**Table 1.** Characteristics of patients who required sports-related emergency transport in Osaka City (2013–2015)

*N* = 2,642	*n* (%)	
Male	2,087	(79.0%)
Age, years		
≤11	219	(8.3%)
12–14	535	(20.2%)
15–17	418	(15.8%)
18–30	672	(25.4%)
31–40	290	(11.0%)
41–60	365	(13.8%)
>60	143	(5.4%)
Time of occurrence		
0:00–5:59	28	(1.1%)
6:00–11:59	616	(23.3%)
12:00–17:59	1,428	(54.0%)
18:00–23:59	570	(21.6%)
Weekday	1,124	(42.5%)
Season		
Spring (March∼May)	626	(23.7%)
Summer (June∼August)	736	(27.9%)
Autumn (September∼November)	796	(30.1%)
Winter (December∼February)	484	(18.3%)
Year		
2013	837	(31.7%)
2014	859	(32.5%)
2015	946	(35.8%)
Location		
Athletic field/stadium	1,159	(43.9%)
School	753	(28.5%)
Park	185	(7.0%)
Street	87	(3.3%)
Pool/waterfront	15	(0.6%)
Other	443	(16.8%)
Cause of accident		
External injury	2,367	(89.6%)
Fall	896	(33.9%)
Collision/Strike	609	(23.1%)
Ball/Flying object	277	(10.5%)
Non-contact	192	(7.3%)
Drowning	2	(0.1%)
Unknown	391	(14.8%)
Illness	275	(10.4%)

Table 2 shows the age-categories of patients according to the type of sports. Overall, sports-related emergency transport was required for a minimum of 80 types of sports. The number of accidents during ball games accounted for the majority of all cases (71.5%, 1,888/2,642). The 10 leading sports considering the number of accidents were baseball (*n* = 380), soccer (*n* = 368), futsal (*n* = 208), basketball (*n* = 194), rugby (*n* = 169), softball (*n* = 139), long-distance running (*n* = 116), volleyball (*n* = 115), tennis (*n* = 84), and judo (*n* = 71). Among these types of sports, the proportion of children and adolescents (age ≤18 years) was relatively high in soccer (79.3%) and rugby (78.1%). In contrast, in tennis and long-distance running, more than half of the accidents (54.8% for tennis and 51.7% for long-distance running) occurred in middle-aged and elderly people (age ≥41 years). The detailed age categories according to the type of sports are also shown in eTable 1.

**Table 2.** Age categories of patients according to the type of sports

		Age, years						Total

		≤18		19–40		≥41		
		*n* (%)		*n* (%)		*n* (%)		
Athletics, jogging	Long-distance running (5000 m∼)	7	(6.0%)	49	(42.2%)	60	(51.7%)	116
	Jogging	16	(57.1%)	5	(17.9%)	7	(25.0%)	28
	Relay	10	(58.8%)	4	(23.5%)	3	(17.6%)	17
	Sprinting (∼400 m)	9	(52.9%)	4	(23.5%)	4	(23.5%)	17
	High jump	10	(76.9%)	3	(23.1%)	0	(0.0%)	13
	Hurdles	12	(100.0%)	0	(0.0%)	0	(0.0%)	12
	Long jump	7	(100.0%)	0	(0.0%)	0	(0.0%)	7
	Road relay	1	(33.3%)	1	(33.3%)	1	(33.3%)	3
	Triple jump	3	(100.0%)	0	(0.0%)	0	(0.0%)	3
	Middle-distance running (800–5000 m)	2	(100.0%)	0	(0.0%)	0	(0.0%)	2

Swimming, water sports	Swimming	3	(27.3%)	3	(27.3%)	5	(45.5%)	11
	Rowing	0	(0.0%)	2	(100.0%)	0	(0.0%)	2
	Diving	3	(100.0%)	0	(0.0%)	0	(0.0%)	3
	Windsurfing	0	(0.0%)	1	(100.0%)	0	(0.0%)	1

Gymnastics	Floor exercises	18	(69.2%)	6	(23.1%)	2	(7.7%)	26
	Horizontal bar	11	(78.6%)	3	(21.4%)	0	(0.0%)	14
	Trampoline	9	(81.8%)	2	(18.2%)	0	(0.0%)	11
	Vault	6	(75.0%)	2	(25.0%)	0	(0.0%)	8
	Baton twirling	2	(100.0%)	0	(0.0%)	0	(0.0%)	2
	Rings	1	(100.0%)	0	(0.0%)	0	(0.0%)	1
	Rhythmic gymnastics	1	(100.0%)	0	(0.0%)	0	(0.0%)	1

Martial arts	Judo	47	(66.2%)	19	(26.8%)	5	(7.0%)	71
	Karate	21	(51.2%)	14	(34.1%)	6	(14.6%)	41
	Kendo	12	(42.9%)	11	(39.3%)	5	(17.9%)	28
	Mixed martial arts	2	(8.7%)	20	(87.0%)	1	(4.3%)	23
	Boxing	4	(20.0%)	14	(70.0%)	2	(10.0%)	20
	Kick boxing	2	(12.5%)	14	(87.5%)	0	(0.0%)	16
	Nippon Kempo	7	(53.8%)	4	(30.8%)	2	(15.4%)	13
	Professional wrestling	0	(0.0%)	10	(90.9%)	1	(9.1%)	11
	Aikido	1	(11.1%)	3	(33.3%)	5	(55.6%)	9
	Sumo	2	(25.0%)	6	(75.0%)	0	(0.0%)	8
	Taekwondo	4	(80.0%)	1	(20.0%)	0	(0.0%)	5
	Shoot boxing	1	(33.3%)	2	(66.7%)	0	(0.0%)	3
	Iaido	0	(0.0%)	0	(0.0%)	2	(100.0%)	2
	Kung fu	1	(100.0%)	0	(0.0%)	0	(0.0%)	1
	Naginata	1	(100.0%)	0	(0.0%)	0	(0.0%)	1
	Fencing	1	(100.0%)	0	(0.0%)	0	(0.0%)	1
	Wrestling	0	(0.0%)	1	(100.0%)	0	(0.0%)	1
	Self defense	0	(0.0%)	1	(100.0%)	0	(0.0%)	1

Ball games	Baseball	217	(57.1%)	114	(30.0%)	49	(12.9%)	380
	Soccer	292	(79.3%)	61	(16.6%)	15	(4.1%)	368
	Futsal	23	(11.1%)	161	(77.4%)	24	(11.5%)	208
	Basketball	116	(59.8%)	67	(34.5%)	11	(5.7%)	194
	Rugby	132	(78.1%)	31	(18.3%)	6	(3.6%)	169
	Softball	38	(27.3%)	37	(26.6%)	64	(46.0%)	139
	Volleyball	30	(26.1%)	44	(38.3%)	41	(35.7%)	115
	Tennis	23	(27.4%)	15	(17.9%)	46	(54.8%)	84
	Badminton	7	(13.2%)	26	(49.1%)	20	(37.7%)	53
	Table tennis	1	(2.2%)	2	(4.4%)	42	(93.3%)	45
	Field hockey	9	(25.0%)	21	(58.3%)	6	(16.7%)	36
	Handball	23	(76.7%)	6	(20.0%)	1	(3.3%)	30
	American football	2	(10.5%)	17	(89.5%)	0	(0.0%)	19
	Dodge ball	8	(66.7%)	1	(8.3%)	3	(25.0%)	12
	Lacrosse	0	(0.0%)	11	(100.0%)	0	(0.0%)	11
	Ten-pin bowling	0	(0.0%)	2	(22.2%)	7	(77.8%)	9
	Kick baseball	6	(100.0%)	0	(0.0%)	0	(0.0%)	6
	Gateball	0	(0.0%)	0	(0.0%)	4	(100.0%)	4
	Golf	1	(25.0%)	0	(0.0%)	3	(75.0%)	4
	Flying discs	0	(0.0%)	1	(100.0%)	0	(0.0%)	1
	Squash	0	(0.0%)	0	(0.0%)	1	(100.0%)	1

Winter sports	Ice skating	15	(29.4%)	21	(41.2%)	15	(29.4%)	51
	Ice hockey	3	(37.5%)	4	(50.0%)	1	(12.5%)	8
	Snowboarding	2	(40.0%)	3	(60.0%)	0	(0.0%)	5
	Speed skating	2	(100.0%)	0	(0.0%)	0	(0.0%)	2
	Figure skating	0	(0.0%)	1	(100.0%)	0	(0.0%)	1

Dance	Dance	6	(35.3%)	5	(29.4%)	6	(35.3%)	17
	Aerobic dance	0	(0.0%)	0	(0.0%)	7	(100.0%)	7
	Social dancing	0	(0.0%)	0	(0.0%)	5	(100.0%)	5
	Ballet	2	(50.0%)	2	(50.0%)	0	(0.0%)	4
	Cheerleading	2	(100.0%)	0	(0.0%)	0	(0.0%)	2
	Folk dancing	0	(0.0%)	0	(0.0%)	1	(100.0%)	1

Others	Sports day	28	(87.5%)	2	(6.3%)	2	(6.3%)	32
	Weight training	2	(15.4%)	5	(38.5%)	6	(46.2%)	13
	Free climbing	4	(30.8%)	5	(38.5%)	4	(30.8%)	13
	Skateboarding	1	(10.0%)	9	(90.0%)	0	(0.0%)	10
	Roller skating	2	(22.2%)	6	(66.7%)	1	(11.1%)	9
	Horse riding	1	(11.1%)	5	(55.6%)	3	(33.3%)	9
	Cycling	1	(14.3%)	4	(57.1%)	2	(28.6%)	7
	Bouldering	0	(0.0%)	3	(75.0%)	1	(25.0%)	4
	Leapfrog	1	(100.0%)	0	(0.0%)	0	(0.0%)	1

Total		1,237	(46.8%)	897	(34.0%)	508	(19.2%)	2,642

Table 3 shows the number of injuries and illness by diagnosis/symptoms according to the type of sports. Among 2,642 patients, 150 suffered multiple injuries/illnesses in an accident, and a total of 2,453 external injuries and 298 illnesses were documented. Typically, the proportion of illness was high in accidents occurring during long-distance running (93.1%, 108/116). Overall, the leading diagnosis/symptom of external injury was fracture/bone contusion (*n* = 701) and that of illness was heatstroke/dehydration (*n* = 184). Serious acute illness, such as sudden cardiac arrest, accounted for 0.6% (16/2,751) of all cases, and half of them (*n* = 8) occurred during running activities including long-distance running, jogging, and road relay. eTable 2 shows the number of injuries/illness by body part according to the type of sports. Overall, the leading body part with injury was the head (*n* = 555), followed by the face (*n* = 334) and arm (*n* = 239).

**Table 3.** Number of injuries/illness by diagnosis/symptoms according to the type of sports

		External injury											Illness							Total
	
		Fracture/ Bone contusion	Bruise	Dislocation/ Subluxation	Sprain	Laceration	Concussion	Ligament rupture/ Tendon rupture	Muscle strain	Spinal cord injury	Other	Total	Heatstroke/ dehydration	Hyperventilation	Syncope/ Seizure	Sudden cardiac arrest	Diarrhea/ Vomiting	Other	Total	
Athletics, jogging	Long-distance running (5000 m∼)	1	4	0	2	1	0	0	0	0	0	8	78	5	9	4	4	8	108	116
	Jogging	5	1	0	2	2	0	0	0	0	0	10	9	2	1	2	0	4	18	28
	Relay	4	3	1	1	1	1	2	2	0	0	15	1	0	0	0	0	2	3	18
	Sprinting (∼400 m)	7	1	0	0	1	0	2	1	0	0	12	1	1	0	0	1	2	5	17
	High jump	5	7	0	1	2	0	0	0	0	0	15	0	0	0	0	0	0	0	15
	Hurdles	7	3	2	1	0	1	0	0	0	0	14	0	0	0	0	0	0	0	14
	Long jump	5	2	0	0	0	0	0	0	0	0	7	0	0	0	0	0	0	0	7
	Road relay	1	0	0	0	1	0	0	0	0	0	2	0	0	0	2	0	0	2	4
	Triple jump	1	0	0	1	0	0	1	0	0	0	3	0	0	0	0	0	0	0	3
	Middle-distance running (800–5000 m)	0	0	0	0	0	0	0	1	0	0	1	0	1	0	0	0	0	1	2

Swimming, water sports	Swimming	1	0	4	0	0	0	0	0	0	2	7	2	0	0	1	0	1	4	11
	Rowing	0	2	0	1	0	0	0	0	0	0	3	0	0	0	0	0	0	0	3
	Diving	1	2	0	0	0	0	0	0	0	0	3	0	0	0	0	0	0	0	3
	Windsurfing	0	1	0	0	0	0	0	0	0	0	1	0	0	0	0	0	0	0	1

Gymnastics	Floor exercises	11	4	3	4	1	0	1	1	0	1	26	0	0	0	1	0	0	1	27
	Horizontal bar	7	3	0	2	0	1	0	0	0	0	13	0	0	0	0	1	1	2	15
	Trampoline	5	1	1	3	1	0	0	0	0	0	11	0	0	0	0	0	0	0	11
	Vault	4	2	1	1	0	0	0	0	0	0	8	0	0	0	0	0	0	0	8
	Baton twirling	0	0	0	0	0	0	0	0	0	0	0	2	0	0	0	0	0	2	2
	Rings	1	0	0	0	0	0	0	0	0	0	1	0	0	0	0	0	0	0	1
	Rhythmic gymnastics	0	0	1	0	0	0	0	0	0	0	1	0	0	0	0	0	0	0	1

Martial arts	Judo	27	17	12	8	3	6	1	0	0	0	74	0	0	0	0	0	0	0	74
	Karate	8	13	3	1	2	7	1	0	2	2	39	1	0	0	0	0	1	2	41
	Kendo	5	2	0	4	0	1	8	0	0	1	21	2	1	2	0	0	2	7	28
	Mixed martial arts	6	12	0	0	2	4	1	0	0	2	27	0	0	0	0	0	0	0	27
	Boxing	2	7	1	0	1	6	0	0	0	0	17	1	1	0	1	0	0	3	20
	Kick boxing	2	7	4	0	2	3	0	0	0	0	18	0	0	0	0	0	0	0	18
	Nippon Kempo	6	4	1	1	0	0	0	0	1	0	13	0	0	0	0	0	0	0	13
	Professional wrestling	4	2	2	1	2	1	0	0	0	0	12	0	0	0	0	0	0	0	12
	Aikido	2	4	1	2	0	1	0	0	0	0	10	0	0	0	0	0	0	0	10
	Sumo	1	3	1	2	1	0	0	1	0	0	9	0	0	0	0	0	0	0	9
	Taekwondo	2	2	0	1	1	0	0	0	0	0	6	0	0	0	0	0	0	0	6
	Shoot boxing	0	2	0	0	1	0	0	0	0	0	3	0	0	0	0	0	0	0	3
	Iaido	0	0	0	0	1	0	0	0	0	0	1	0	0	0	1	0	0	1	2
	Kung fu	1	0	0	0	0	0	0	0	0	0	1	0	0	0	0	0	0	0	1
	Naginata	0	0	1	0	0	0	0	0	0	0	1	0	0	0	0	0	0	0	1
	Fencing	0	0	0	1	0	0	0	0	0	0	1	0	0	0	0	0	0	0	1
	Wrestling	0	0	0	0	0	0	1	0	0	0	1	0	0	0	0	0	0	0	1
	Self defense	0	0	1	0	0	0	0	0	0	0	1	0	0	0	0	0	0	0	1

Ball games	Baseball	103	149	27	22	42	18	5	6	2	2	376	23	2	0	0	1	6	32	408
	Soccer	120	97	25	25	41	34	7	5	0	6	360	18	0	2	0	2	2	24	384
	Futsal	67	33	30	16	13	11	30	4	1	2	207	0	0	0	2	0	0	2	209
	Basketball	33	47	32	18	17	13	19	3	0	2	184	8	4	0	1	0	2	15	199
	Rugby	40	42	22	9	9	37	2	3	3	2	169	6	0	0	0	0	0	6	175
	Softball	42	48	7	8	10	7	3	5	0	4	134	7	0	1	0	0	2	10	144
	Volleyball	15	16	19	18	10	5	20	4	0	0	107	8	2	0	0	0	1	11	118
	Tennis	18	17	5	8	3	1	17	3	0	0	72	9	1	1	0	1	3	15	87
	Badminton	1	3	2	14	2	1	18	5	0	1	47	1	1	1	0	0	3	6	53
	Table tennis	18	13	1	6	3	0	3	3	0	0	47	1	0	0	0	1	0	2	49
	Field hockey	9	10	1	1	16	0	0	0	0	0	37	0	0	0	0	0	0	0	37
	Handball	7	9	5	5	0	0	4	0	0	0	30	0	0	0	0	0	0	0	30
	American football	6	1	5	3	1	0	0	0	0	1	17	2	0	1	0	0	0	3	20
	Dodge ball	2	4	2	0	2	2	0	0	0	0	12	0	0	0	0	0	0	0	12
	Lacrosse	0	5	0	3	0	1	0	0	0	1	10	2	0	0	0	0	0	2	12
	Ten-pin bowling	7	2	0	0	0	0	0	0	0	0	9	0	0	0	0	0	0	0	9
	Kick baseball	1	2	0	0	1	1	0	0	0	0	5	0	0	1	0	0	0	1	6
	Gateball	2	0	1	0	0	0	0	0	0	0	3	0	0	0	0	0	1	1	4
	Golf	0	0	1	0	1	0	0	0	0	0	2	1	0	0	0	0	1	2	4
	Flying discs	0	0	0	0	0	0	0	0	0	0	0	1	0	0	0	0	0	1	1
	Squash	0	0	0	0	0	0	1	0	0	0	1	0	0	0	0	0	0	0	1

Winter sports	Ice skating	28	16	0	4	2	2	0	0	0	1	53	0	0	0	0	0	0	0	53
	Ice hockey	4	2	2	0	0	1	0	0	0	0	9	0	0	0	0	0	0	0	9
	Snowboarding	5	0	0	0	0	0	0	0	0	0	5	0	0	0	0	0	0	0	5
	Speed skating	0	0	0	0	2	0	0	0	0	0	2	0	0	0	0	0	0	0	2
	Figure skating	0	2	0	0	0	0	0	0	0	0	2	0	0	0	0	0	0	0	2

Dance	Dance	2	2	2	4	0	0	5	0	0	0	15	0	1	0	0	0	1	2	17
	Aerobic dance	3	2	0	1	0	0	0	1	0	0	7	0	0	0	0	0	0	0	7
	Social dancing	0	3	0	0	0	0	0	0	0	0	3	0	0	1	1	0	0	2	5
	Ballet	0	1	2	1	0	0	0	0	0	0	4	0	0	0	0	0	0	0	4
	Cheerleading	1	1	0	0	0	0	0	0	0	0	2	0	0	0	0	0	0	0	2
	Folk dancing	0	0	0	0	0	0	0	0	0	0	0	0	0	0	0	0	1	1	1

Others	Sports day	11	15	0	3	1	4	0	0	0	0	34	0	0	0	0	0	0	0	34
	Weight training	3	4	3	2	0	0	0	0	0	0	12	0	0	0	0	1	0	1	13
	Free climbing	3	1	5	2	1	0	0	0	1	0	13	0	0	0	0	0	0	0	13
	Cycling	1	3	1	2	5	0	0	0	0	0	12	0	0	0	0	0	0	0	12
	Skateboarding	6	0	1	0	3	0	0	0	0	0	10	0	0	0	0	0	0	0	10
	Roller skating	4	3	1	1	0	1	0	0	0	0	10	0	0	0	0	0	0	0	10
	Horse riding	5	3	1	0	0	0	0	0	1	0	10	0	0	0	0	0	0	0	10
	Bouldering	2	0	1	1	0	0	0	0	0	0	4	0	0	0	0	0	0	0	4
	Leapfrog	0	0	0	0	0	0	0	0	0	1	1	0	0	0	0	0	0	0	1

Total		701	667	244	217	211	171	152	48	11	31	2,453	184	22	20	16	12	44	298	2,751

## DISCUSSION

Using the exhaustive ambulance records in Osaka City, we provided detailed characteristics of patients who required sports-related emergency transport. Our results confirmed that approximately 800–900 cases were transported to hospital by the EMS owing to sports-related accidents each year. Although they represented a small subset of the overall burden on emergency transport in this population (0.4%), they occurred during participation in a wide variety of sports. We understand that this is the first statistical report to depict the epidemiological features of sports-related emergency transport at the population-level. Our findings provide further essential information for developing preventative strategies to inform administrative officials of sports events, trainers, athletes, and recreational sports participants, as well as to draw the attention of medical staff to consider in greater depth the mechanism and treatment of sports-related accidents.

In Osaka City, the need for emergency transport was documented for at least 80 types of sports, and the number of patients ranged from 1 to 380 by the type of sport. Such differences in the number of patients may be affected by the level of risk, as well as the popularity of specific sports, population composition, and other environmental factors among the study population. As this study was based on the population-based ambulance records, our findings reflect the situation of the general population living in Osaka City, and the pattern of accidents could differ in other communities or nations. However, our results suggest that any type of sport has potential risk of serious injury and illness that may require emergency transport, conveying an important message for all people engaging in sports activities.

The present study provided several important features of sports-related emergency transport among the general population. First, fracture/bone contusion was the leading issue requiring emergency transport; this finding is consistent with that of the previous study that focused on the epidemiology of emergency transport among collegiate and high-school student-athletes,^3^ and with the study that showed that fractures were a frequent diagnosis in athletes presenting to the emergency department.^4^ Although strains and sprains are common injuries in athletes across competition levels,^5^^–^^7^ they might not be typically considered conditions necessitating emergency transport. Second, the head and face were the commonly injured body parts, consistent with previous findings.^3^ Head and face injuries require an advanced level of judgment for determining the severity of injury because these regions surround the central nervous system. Third, the majority of sports-related emergency transport cases were observed in men. Although these differences between the sexes were also observed in competitive athletes previously in other countries,^2^^,^^3^ the exact reasons for the disparities could not be determined from our data; this may be owing to variations between men and women considering the participation rates and duration or level of exertion during each type of sport.

Our findings also demonstrated that the characteristics of accidents widely varied by the type of sports. For example, unlike with other type of sports, most accidents during running activities, such as long-distance running, jogging, and road relay, were ascribed to illness rather than external injuries. Importantly, half of sudden cardiac arrest cases, the most serious reported acute illness, occurred during these types of sports. In Japan, participation in jogging or running activities has recently gained popularity, with the number of participants constantly increasing over the past 15 years.^8^ Vigorous and long-time physical exertion as in long-distance running can trigger the onset of acute myocardial infarction, particularly in people who are typically sedentary.^9^ Similarly, jogging or running accounted for over 25% of exercise-related sudden cardiac arrests among middle-aged patients in a study in the United States.^10^ Therefore, it is important to disseminate public-access defibrillation programs to staff, trainers, and participants of long-distance running events.

### Limitations

This study has several inherent limitations. First, information regarding in-hospital outcomes and treatment of emergency patients after hospital arrival was not available in this registry. In addition, we did not obtain information about several background factors that could have influenced the occurrence of accidents, such as the patient’s condition, medical history, medication, lifestyle, exercise intensity, and the frequency of habitual training. Second, as the present study was based on the ambulance records, only emergency patients who were transported to hospital by EMS personnel were enrolled. Therefore, most of our study subjects may have experienced serious acute injuries/illnesses. Third, the absolute number of individuals engaged in each sport in Osaka City was not available. Therefore, we were not able to estimate the incidence rate of the issues. Finally, our study area was limited to a single densely populated area in Japan. Therefore, results may not be generalizable to other areas worldwide. In particular, large-scale marathon events in which more than 10,000 general citizens participate are held every year in Osaka, such as the Osaka Marathon, the Yodogawa Kanpei Marathon, and the Osaka Yodo-river Citizens Marathon. Therefore, injuries and illnesses related to long distance running may be more likely to occur in Osaka City. Further investigations using data from other communities worldwide are needed to confirm our findings.

### Conclusion

Characteristics of accidents occurring during sports activity widely varied by the type of sports. Measures to prevent serious accidents during sports activities should be established based on the patient characteristics for each type of sport.

## APPENDIX A. SUPPLEMENTARY DATA

eTable 1. Age categories of patients according to the type of sports

eTable 2. Number of injuries/illness by body part according to the type of sports
