# Supplementary material for: Characteristics of Sports-Related Emergency Transport: A Population-Based Descriptive Study in Osaka City
Source: J Epidemiol. 2020 Jun 5;30(6):268–75. doi: 10.2188/jea.JE20190019 (PMC7217686; doi:10.2188/jea.JE20190019)
Supplement: Supplementary file 1 [file je-30-268-s001.pdf]

eTable 1. Age categories of patients according to the type of sports

		Age, years																		Total
		≤5	6–9	10	11	12	13	14	15	16	17	18	19	20–23	24–30	31–40	41–50	51–60	>60	
Athletics, jogging	Long-distance running (5000 m~)	0	0	0	0	0	2	1	1	1	1	1	1	7	14	27	37	11	12	116
	Jogging	0	1	0	1	1	1	3	2	6	1	0	0	2	0	3	5	1	1	28
	Relay	0	0	0	1	0	2	0	2	3	2	0	0	2	0	2	2	1	0	17
	Sprinting (~400 m)	0	0	0	1	0	4	0	3	1	0	0	3	1	0	0	3	0	1	17
	High jump	0	0	0	2	0	4	3	1	0	0	0	0	3	0	0	0	0	0	13
	Hurdles	0	0	1	0	1	2	4	0	1	2	1	0	0	0	0	0	0	0	12
	Long jump	0	0	0	1	1	1	3	0	0	0	1	0	0	0	0	0	0	0	7
	Road relay	0	0	0	0	0	0	0	1	0	0	0	0	1	0	0	0	1	0	3
	Triple jump	0	0	0	0	0	0	0	0	1	2	0	0	0	0	0	0	0	0	3
	Middle-distance running (800–5000 m)	0	0	0	0	0	1	0	0	0	1	0	0	0	0	0	0	0	0	2
Swimming, water sports	Swimming	0	0	1	0	0	0	0	0	1	1	0	0	1	2	0	0	1	4	11
	Rowing	0	0	0	0	0	0	0	0	0	0	0	1	0	0	1	0	0	0	2
	Diving	0	0	0	0	1	0	1	1	0	0	0	0	0	0	0	0	0	0	3
	Windsurfing	0	0	0	0	0	0	0	0	0	0	0	1	0	0	0	0	0	0	1
Gymnastics	Floor exercises	1	0	0	1	1	5	3	2	1	4	0	2	1	3	0	0	1	1	26
	Horizontal bar	0	4	1	2	2	0	1	0	1	0	0	2	1	0	0	0	0	0	14
	Trampoline	0	2	1	0	2	0	1	1	2	0	0	1	1	0	0	0	0	0	11
	Vault	0	1	1	1	0	1	0	1	0	1	0	1	0	1	0	0	0	0	8
	Baton twirling	0	0	0	0	1	1	0	0	0	0	0	0	0	0	0	0	0	0	2
	Rings	0	0	0	0	0	0	0	0	1	0	0	0	0	0	0	0	0	0	1
	Rhythmic gymnastics	0	0	0	0	0	0	1	0	0	0	0	0	0	0	0	0	0	0	1
Martial arts	Judo	0	2	3	2	4	4	10	6	10	4	2	3	5	7	4	3	1	1	71
	Karate	1	4	1	1	0	1	1	5	3	3	1	4	6	0	4	5	0	1	41
	Kendo	0	1	4	1	2	2	0	0	1	0	1	0	3	4	4	2	3	0	28
	Mixed martial arts	0	0	0	0	0	0	1	0	0	0	1	2	5	8	5	1	0	0	23
	Boxing	0	0	0	0	0	0	0	1	0	1	2	1	6	6	1	1	0	1	20
	Kick boxing	0	0	0	0	0	0	1	0	0	0	1	0	6	6	2	0	0	0	16
	Nippon Kempo	0	1	0	0	0	0	0	0	4	1	1	1	2	1	0	2	0	0	13
	Professional wrestling	0	0	0	0	0	0	0	0	0	0	0	0	0	3	7	1	0	0	11
	Aikido	0	0	0	0	0	0	0	0	1	0	0	2	0	0	1	3	0	2	9
	Sumo	0	0	0	0	0	0	0	0	0	2	0	0	1	2	3	0	0	0	8
	Taekwondo	0	0	0	0	0	1	2	0	0	1	0	0	1	0	0	0	0	0	5
	Shoot boxing	0	0	0	0	0	0	0	1	0	0	0	0	0	1	1	0	0	0	3
	Iaido	0	0	0	0	0	0	0	0	0	0	0	0	0	0	0	0	0	2	2
	Kung fu	0	0	0	1	0	0	0	0	0	0	0	0	0	0	0	0	0	0	1
	Naginata	0	0	0	0	0	0	0	1	0	0	0	0	0	0	0	0	0	0	1
	Fencing	0	0	0	0	0	0	0	0	1	0	0	0	0	0	0	0	0	0	1
	Wrestling	0	0	0	0	0	0	0	0	0	0	0	0	0	0	1	0	0	0	1
	Self defense	0	0	0	0	0	0	0	0	0	0	0	0	0	1	0	0	0	0	1
Ball games	Baseball	0	14	11	19	29	37	41	18	23	17	8	8	31	38	37	30	5	14	380
	Soccer	0	13	19	18	35	54	51	34	36	19	13	4	12	28	17	10	4	1	368
	Futsal	0	6	0	3	2	0	2	1	3	2	4	3	18	76	64	18	6	0	208
	Basketball	0	2	5	10	9	19	18	13	16	15	9	10	17	21	19	8	2	1	194
	Rugby	0	2	0	0	15	33	37	15	14	12	4	4	17	9	1	4	2	0	169
	Softball	0	5	4	6	3	4	4	4	5	3	0	0	10	4	23	34	16	14	139
	Volleyball	0	1	0	1	2	6	4	1	7	4	4	1	4	12	27	26	13	2	115
	Tennis	0	0	1	0	1	2	3	5	9	1	1	2	5	6	2	19	14	13	84
	Badminton	0	0	0	0	0	2	1	2	1	1	0	3	6	8	9	12	6	2	53

	Table tennis	0	0	0	0	0	0	0	0	1	0	0	0	0	2	0	1	5	36	45
	Field hockey	0	0	0	2	2	0	1	2	1	1	0	6	11	3	1	2	0	4	36
	Handball	0	0	0	0	0	1	4	2	11	4	1	1	1	2	2	1	0	0	30
	American football	0	0	0	0	0	0	0	0	1	1	0	0	9	6	2	0	0	0	19
	Dodge ball	0	4	2	0	1	0	0	1	0	0	0	1	0	0	0	3	0	0	12
	Lacrosse	0	0	0	0	0	0	0	0	0	0	0	3	6	1	1	0	0	0	11
	Ten-pin bowling	0	0	0	0	0	0	0	0	0	0	0	0	0	1	1	1	1	5	9
	Kick baseball	0	3	1	1	1	0	0	0	0	0	0	0	0	0	0	0	0	0	6
	Gateball	0	0	0	0	0	0	0	0	0	0	0	0	0	0	0	0	0	4	4
	Golf	0	1	0	0	0	0	0	0	0	0	0	0	0	0	0	0	1	2	4
	Flying discs	0	0	0	0	0	0	0	0	0	0	0	0	1	0	0	0	0	0	1
	Squash	0	0	0	0	0	0	0	0	0	0	0	0	0	0	0	0	1	0	1
Winter sports	Ice skating	0	7	1	1	4	0	1	0	0	0	1	1	5	7	8	9	3	3	51
	Ice hockey	0	0	0	0	0	0	2	0	0	1	0	2	1	0	1	0	0	1	8
	Snowboarding	0	0	0	0	0	0	0	1	0	0	1	0	0	3	0	0	0	0	5
	Speed skating	0	0	0	0	0	0	0	0	0	1	1	0	0	0	0	0	0	0	2
	Figure skating	0	0	0	0	0	0	0	0	0	0	0	0	1	0	0	0	0	0	1
Dance	Dance	0	0	0	0	0	1	1	1	1	2	0	1	1	3	0	2	2	2	17
	Aerobic dance	0	0	0	0	0	0	0	0	0	0	0	0	0	0	0	2	1	4	7
	Social dancing	0	0	0	0	0	0	0	0	0	0	0	0	0	0	0	0	1	4	5
	Ballet	0	0	0	0	0	1	0	0	0	0	1	0	0	2	0	0	0	0	4
	Cheerleading	0	1	0	0	0	0	0	0	1	0	0	0	0	0	0	0	0	0	2
	Folk dancing	0	0	0	0	0	0	0	0	0	0	0	0	0	0	0	0	0	1	1
Others	Sports day	0	1	1	6	4	0	10	2	1	2	1	0	0	1	1	2	0	0	32
	Weight training	0	0	0	0	0	0	1	0	1	0	0	0	1	3	1	5	0	1	13
	Free climbing	0	0	0	0	0	0	0	1	1	0	2	0	0	3	2	1	2	1	13
	Skateboarding	0	1	0	0	0	0	0	0	0	0	0	1	1	6	1	0	0	0	10
	Roller skating	0	0	0	0	1	0	0	0	0	0	1	0	2	3	1	1	0	0	9
	Horse riding	0	0	0	0	1	0	0	0	0	0	0	0	5	0	0	0	1	2	9
	Cycling	0	0	0	0	0	0	0	0	1	0	0	0	1	1	2	1	1	0	7
	Bouldering	0	0	0	0	0	0	0	0	0	0	0	0	1	1	1	1	0	0	4
	Leapfrog	0	0	0	0	0	0	0	0	0	0	1	0	0	0	0	0	0	0	1
	Total	2	77	58	82	126	192	217	132	173	113	65	76	222	309	290	258	107	143	2,642

eTable 2. Number of injuries/illness by body part according to the type of sports

		External injury															Illness						Total
		Face	Head	Neck	Trunk	Shoulder	Arm	Elbow	Wrist/ Hand	Hip joint/ Groin	Leg	Knee	Achilles' tendon	Ankle joint/ Foot	Unclassifiable	Total	Respiratory	Neurologic/ Mental	Cardiovascular	Gastrointestinal	Other	Total	
Athletics, jogging	Long-distance running (5000 m~)	0	3	0	1	0	0	0	0	1	2	1	0	0	0	8	4	5	7	20	72	108	116
	Jogging	1	1	0	0	0	1	0	1	0	3	2	0	1	0	10	1	2	2	3	10	18	28
	Relay	1	3	0	2	3	1	0	0	0	2	0	2	1	0	15	0	0	0	0	3	3	18
	Sprinting (~400 m)	0	0	0	5	0	1	1	0	0	3	0	2	0	0	12	0	1	0	2	2	5	17
	High jump	2	1	0	2	1	3	0	0	0	3	3	0	0	0	15	0	0	0	0	0	0	15
	Hurdles	0	2	0	2	1	3	3	0	0	1	0	0	2	0	14	0	0	0	0	0	0	14
	Long jump	0	1	0	1	0	1	1	1	0	0	1	0	1	0	7	0	0	0	0	0	0	7
	Road relay	1	0	0	0	0	1	0	0	0	0	0	0	0	0	2	0	0	2	0	0	2	4
	Triple jump	0	0	0	0	0	0	0	0	0	1	0	1	1	0	3	0	0	0	0	0	0	3
	Middle-distance running (800–5000 m)	0	0	0	0	0	0	0	0	0	1	0	0	0	0	1	0	1	0	0	0	1	2
Swimming, water sports	Swimming	0	0	0	0	4	0	0	0	1	0	0	0	0	2	7	0	0	2	1	1	4	11
	Rowing	1	1	0	1	0	0	0	0	0	0	0	0	0	0	3	0	0	0	0	0	0	3
	Diving	1	1	1	0	0	0	0	0	0	0	0	0	0	0	3	0	0	0	0	0	0	3
	Windsurfing	0	1	0	0	0	0	0	0	0	0	0	0	0	0	1	0	0	0	0	0	0	1
Gymnastics	Floor exercises	3	4	2	3	0	3	2	0	1	3	2	1	2	0	26	0	0	1	0	0	1	27
	Horizontal bar	1	2	1	1	1	4	2	0	0	0	0	0	1	0	13	0	0	1	1	0	2	15
	Trampoline	1	1	3	1	0	3	0	0	0	0	2	0	0	0	11	0	0	0	0	0	0	11
	Vault	0	0	0	2	0	3	1	0	0	0	2	0	0	0	8	0	0	0	0	0	0	8
	Baton twirling	0	0	0	0	0	0	0	0	0	0	0	0	0	0	0	0	0	0	2	0	2	2
	Rings	0	0	0	0	0	1	0	0	0	0	0	0	0	0	1	0	0	0	0	0	0	1
	Rhythmic gymnastics	0	0	0	0	0	0	1	0	0	0	0	0	0	0	1	0	0	0	0	0	0	1
Martial arts	Judo	1	20	5	4	14	3	8	2	1	4	3	1	8	0	74	0	0	0	0	0	0	74
	Karate	9	11	3	2	2	1	0	1	2	1	2	1	4	0	39	0	0	1	0	1	2	41
	Kendo	0	3	1	3	0	1	0	1	0	0	2	8	2	0	21	0	2	0	2	3	7	28
	Mixed martial arts	8	11	0	2	2	0	0	1	0	2	0	1	0	0	27	0	0	0	0	0	0	27
	Boxing	6	9	0	0	2	0	0	0	0	0	0	0	0	0	17	1	1	1	0	0	3	20
	Kick boxing	4	3	0	2	1	0	1	2	0	3	0	0	2	0	18	0	0	0	0	0	0	18
	Nippon Kempo	0	1	1	2	3	3	3	0	0	0	0	0	0	0	13	0	0	0	0	0	0	13
	Professional wrestling	2	2	0	2	1	1	1	0	0	2	1	0	0	0	12	0	0	0	0	0	0	12
	Aikido	0	3	1	1	1	0	1	0	0	0	1	0	2	0	10	0	0	0	0	0	0	10
	Sumo	1	0	0	2	1	0	0	0	0	2	3	0	0	0	9	0	0	0	0	0	0	9
	Taekwondo	1	1	0	1	0	2	0	0	0	0	1	0	0	0	6	0	0	0	0	0	0	6
	Shoot boxing	2	0	0	1	0	0	0	0	0	0	0	0	0	0	3	0	0	0	0	0	0	3
	Iaido	0	0	0	0	0	0	0	1	0	0	0	0	0	0	1	0	0	1	0	0	1	2
	Kung fu	0	0	0	0	0	1	0	0	0	0	0	0	0	0	1	0	0	0	0	0	0	1
	Naginata	0	0	0	0	1	0	0	0	0	0	0	0	0	0	1	0	0	0	0	0	0	1
	Fencing	0	0	0	1	0	0	0	0	0	0	0	0	0	0	1	0	0	0	0	0	0	1
	Wrestling	0	0	0	0	0	0	0	0	0	0	0	1	0	0	1	0	0	0	0	0	0	1
	Self defense	0	0	0	0	0	0	0	0	0	0	1	0	0	0	1	0	0	0	0	0	0	1

Ball games	Baseball	104	84	9	26	31	30	4	15	5	26	24	1	17	0	376	2	2	1	5	22	32	408
	Soccer	40	97	5	23	23	62	13	16	3	34	17	6	18	3	360	1	1	0	6	16	24	384
	Futsal	16	28	2	15	15	18	10	15	2	23	13	28	22	0	207	0	0	2	0	0	2	209
	Basketball	15	55	2	5	17	15	5	16	2	7	13	18	13	1	184	0	4	1	4	6	15	199
	Rugby	16	69	5	7	19	9	7	10	0	9	4	2	9	3	169	0	0	0	1	5	6	175
	Softball	40	29	1	6	6	5	1	17	1	11	5	4	7	1	134	3	0	0	0	7	10	144
	Volleyball	10	15	1	7	7	3	3	12	0	7	7	20	15	0	107	1	2	0	0	8	11	118
	Tennis	4	12	0	0	6	6	0	2	1	11	5	17	8	0	72	1	1	0	2	11	15	87
	Badminton	3	2	0	1	1	0	0	1	0	8	9	18	4	0	47	0	1	2	0	3	6	53
	Table tennis	3	6	0	15	1	9	0	1	2	5	1	3	1	0	47	0	0	0	1	1	2	49
	Field hockey	19	8	0	1	3	2	0	1	1	0	2	0	0	0	37	0	0	0	0	0	0	37
	Handball	2	4	1	2	4	4	1	0	0	1	4	4	3	0	30	0	0	0	0	0	0	30
	American football	0	2	0	2	4	2	0	3	0	0	2	0	2	0	17	0	1	0	1	1	3	20
	Dodge ball	1	7	0	0	1	1	0	1	1	0	0	0	0	0	12	0	0	0	0	0	0	12
	Lacrosse	2	3	1	1	0	0	1	0	0	0	1	0	1	0	10	0	0	0	0	2	2	12
	Ten-pin bowling	0	2	0	0	0	3	0	1	0	1	0	0	2	0	9	0	0	0	0	0	0	9
	Kick baseball	1	2	0	0	0	1	0	0	0	0	1	0	0	0	5	0	0	1	0	0	1	6
	Gateball	0	0	0	0	1	0	0	0	2	0	0	0	0	0	3	0	0	0	0	1	1	4
	Golf	1	0	0	0	1	0	0	0	0	0	0	0	0	0	2	0	0	0	0	2	2	4
	Flying discs	0	0	0	0	0	0	0	0	0	0	0	0	0	0	0	0	0	0	0	1	1	1
	Squash	0	0	0	0	0	0	0	0	0	0	0	1	0	0	1	0	0	0	0	0	0	1
Winter sports	Ice skating	3	16	0	2	2	9	0	1	2	12	1	0	5	0	53	0	0	0	0	0	0	53
	Ice hockey	2	2	0	0	3	0	0	0	0	1	0	0	1	0	9	0	0	0	0	0	0	9
	Snowboarding	0	0	0	0	0	4	0	0	0	0	0	0	1	0	5	0	0	0	0	0	0	5
	Speed skating	1	0	0	0	0	0	0	0	0	1	0	0	0	0	2	0	0	0	0	0	0	2
	Figure skating	0	0	0	2	0	0	0	0	0	0	0	0	0	0	2	0	0	0	0	0	0	2
Dance	Dance	0	2	0	1	0	1	0	0	0	0	5	4	2	0	15	0	1	0	0	1	2	17
	Aerobic dance	0	0	0	1	0	2	0	1	0	1	1	0	1	0	7	0	0	0	0	0	0	7
	Social dancing	0	3	0	0	0	0	0	0	0	0	0	0	0	0	3	0	0	2	0	0	2	5
	Ballet	0	1	0	0	1	0	0	0	0	0	2	0	0	0	4	0	0	0	0	0	0	4
	Cheerleading	0	1	0	0	1	0	0	0	0	0	0	0	0	0	2	0	0	0	0	0	0	2
	Folk dancing	0	0	0	0	0	0	0	0	0	0	0	0	0	0	0	0	0	1	0	0	1	1
Others	Sports day	1	13	2	3	1	7	4	0	0	2	0	0	1	0	34	0	0	0	0	0	0	34
	Weight training	1	1	0	5	3	1	0	0	0	0	0	0	1	0	12	0	0	0	1	0	1	13
	Free climbing	0	0	1	2	3	0	1	0	0	1	2	0	2	1	13	0	0	0	0	0	0	13
	Cycling	2	1	1	2	3	1	0	0	0	0	0	0	0	2	12	0	0	0	0	0	0	12
	Skateboarding	0	2	0	0	2	1	0	0	1	3	0	0	1	0	10	0	0	0	0	0	0	10
	Roller skating	0	3	0	0	1	4	1	0	0	0	0	0	1	0	10	0	0	0	0	0	0	10
	Horse riding	1	0	0	4	2	1	1	0	0	1	0	0	0	0	10	0	0	0	0	0	0	10
	Bouldering	0	0	0	0	1	1	0	0	0	0	1	0	1	0	4	0	0	0	0	0	0	4
	Leapfrog	0	0	0	0	0	0	0	0	0	1	0	0	0	0	1	0	0	0	0	0	0	1
	Total	334	555	49	177	201	239	77	123	29	199	147	144	166	13	2453	14	25	28	52	179	298	2,751
